# Two-Year Bonding Performances of a Universal Adhesive After Dentin Pre-Treatment With Two-Step Silver-Containing Solutions

**DOI:** 10.3290/j.jad.c_2351

**Published:** 2025-11-18

**Authors:** Carlo D’Alessandro, Uros Josic, Tatjana Maravic, Diego D’Urso, Vittorio Checchi, Annalisa Mazzoni, Lorenzo Breschi, Claudia Mazzitelli

**Affiliations:** a Carlo D’Alessandro PhD student, Department of Biomedical and Neuromotor Sciences (DIBINEM), Alma Mater Studiorum, University of Bologna, Bologna, Italy. Formal analysis, investigation, methodology, writing – original draft.; b Uros Josic Research fellow, Department of Biomedical and Neuromotor Sciences (DIBINEM), Alma Mater Studiorum, University of Bologna, Bologna, Italy. Investigation, methodology, validation, writing – review and editing.; c Tatjana Maravic Junior Assistant Professor, Department of Biomedical and Neuromotor Sciences (DIBINEM), Alma Mater Studiorum, University of Bologna, Bologna, Italy. Methodology, validation, writing – review, and editing.; d Diego D’Urso PhD Student, Department of Biomedical and Neuromotor Sciences (DIBINEM), Alma Mater Studiorum, University of Bologna, Bologna, Italy. Investigation, methodology, validation. writing – original draft.; e Vittorio Checchi Associate Professor, Department of Surgery, Medicine, Dentistry and Morphological Sciences, University of Modena and Reggio Emilia, Modena, Italy. Investigation, writing – review and editing.; f Annalisa Mazzoni Full Professor, Department of Biomedical and Neuromotor Sciences (DIBINEM), Alma Mater Studiorum, University of Bologna, Bologna, Italy. Supervision, Writing – review & editing.; g Lorenzo Breschi Full Professor, Department of Biomedical and Neuromotor Sciences (DIBINEM), Alma Mater Studiorum, University of Bologna, Bologna, Italy. Conceptualization, supervision, writing – review and editing.; h Claudia Mazzitelli Assistant Professor, Department of Biomedical and Neuromotor Sciences (DIBINEM), Alma Mater Studiorum, University of Bologna, Bologna, Italy. Conceptualization, project administration, supervision, writing – review and editing.

**Keywords:** silver diamine fluoride, silver fluoride, potassium iodide, universal adhesive, composite resin, bond strength, MMPs, aging

## Abstract

**Purpose:**

To evaluate the effects of dentin pre-treatment with an ammonia- and water-based 2-step silver-containing solutions on the microtensile bond strength (µTBS), chemo-morphological characterization (SEM/EDS), and matrix metalloproteinases (MMPs) activity of a universal adhesive after 2 years of artificial aging.

**Materials and Methods:**

Mid-coronal dentin surfaces of sound human molars (N = 60) were exposed and grouped according to the following pre-treatment and universal adhesive (Zipbond Universal, SDI) etching mode (n = 10): G1) Zipbond in the self-etch mode (control SE group, ZSE); G2) Riva Star (SDI) and ZSE; G3) Riva Star Aqua (SDI) and ZSE; G4) Zipbond in the etch-and-rinse mode (control ER group, ZER); G5) Riva Star and ZER; G6) Riva Star Aqua and ZER. µTBS test, SEM/EDS, and *in-situ* zymography analyses were conducted at baseline (T0) and after 2 years (T2) of storage in artificial saliva. Data were statistically analyzed (P = 0.05).

**Results:**

Experimental groups exhibited significantly lower bond strength compared to control groups (Control>Riva Star=Riva Star Aqua; P >0.05). Artificial aging reduced bond strength values in all the groups (P <0.05). ER groups provided higher bond strength than SE ones (P <0.05). No silver ions were detected at T0 and T2 in the adhesive interfaces of specimens treated with both silver-containing solutions (SEM/EDS). Riva Star significantly decreased the MMPs activity (P <0.05) compared to control and Riva Star Aqua (P <0.05).

**Conclusion:**

Dentin pre-treatment with the tested two-step silver-containing solutions impaired the 2-year bonding performance of composite resin restorations placed with a universal adhesive. The detected level of endogenous enzymatic activity was found to be product-dependent.

In the past decades, restorative dentistry has increasingly focused on minimally invasive approaches to ensure long-term clinical outcomes.^42^ Therefore, researchers have investigated different techniques and explored a range of therapeutic materials with antimicrobial and remineralizing properties.^40,55^

Initially introduced as a cavity varnish,^58^ silver diamine fluoride (SDF) has spread globally as a non-invasive approach to manage carious lesions.^57^ Leveraging the bioactivity of silver ions,^52^ its application has been extended to various indications, including as a desensitizing agent,^9^ root canal irrigant,^48^ and cavity cleanser.^29^ Notably, in 2021, the World Health Organization (WHO) recognized SDF as one of the most effective, safe, manageable, non-invasive, and affordable agents for both adult and paediatric patients.^59^ However, the downside of SDF solutions is the dark staining of tooth substrates following their application.^3^

Recently, the implementation of potassium iodide (KI) solution as a second clinical step after the application of SDF has gained attention due to its ability to mitigate tooth discoloration.^3^ These two-step silver-containing solutions aim to minimize dark staining while preserving the bioactive properties.^23^

There is a general consensus that resin–dentin bonds created with adhesive systems deteriorate over time,^7^ and host-derived dentin proteases, especially the endogenous matrix metalloprotein-ases (MMPs), significantly contribute to hybrid layer (HL) degradation.^36^ Given their aforementioned potential to hinder MMP activity, these silver-containing products have also been proposed as therapeutic agents to preserve HL integrity.^39^ As dentin surface pre-treatments, they may exert an important role in creating a stable and durable HL, enhancing dentin bond strength and restorations’ longevity.^19^

Although widely employed in preventive and restorative dentistry, there is limited evidence regarding the impact of two-step silver-containing solutions on dentin bonding performance in direct composite restorations, particularly in association with universal adhesive systems.^25^ Simulating intraoral aging is crucial for predicting the behavior of materials and bonding durability over time.^51^ However, most of the existing evidence regarding the effects of silver-containing products on restorative materials has only evaluated initial bond strength, neglecting the impact of aging on HL degradation.^20^ A limited number of studies have investigated dentin bond strength over time,^18,28,16,41,12^ with just one article comparing an ammonia-based and a novel water-based silver-containing solutions after 1 year of artificial storage.^12^ An extended observation period was warranted to obtain more consistent data on the long-term effects of these therapeutic agents in terms of bond strength and MMPs activity.

Therefore, this *in-vitro* study aimed to investigate the influence of dentin surface pre-treatment with two-step silver-containing products, an ammonia-based (Riva Star, SDI, Bayswater VIC, Aus-tralia) and a water-based (Riva Star Aqua, SDI) solution, respectively, on the bonding performances and MMPs activity of a universal adhesive at baseline (T0) and after 2 years (T2) of artificial aging. Specifically, the following working hypotheses were tested: (1) dentin pre-treatment with two-step silver-containing solutions influences microtensile bond strength of composite restorations placed with a universal adhesive; (2) dentin pre-treatment influences the endogenous enzymatic activity; and (3) artificial aging has an impact on microtensile bond strength and endogenous enzymatic activity.

## MATERIALS AND METHODS

### Teeth Collection

Sixty sound human molars, freshly extracted from anonymous individuals after their signed informed consent for research purposes, were obtained according to the protocol (protocol N°: 71/2019/OSS/AUSLBO) approved by the Ethics Committee of the University of Bologna (Italy) and used for this study. A pilot study was conducted to determine the proper sample size for the testing procedures conducted in the present investigation with an alpha error of 0.05 and a power of 90% (G*Power 3.1).^17^

### Microtensile Bond Strength (µTBS) Test

The specimens’ preparation and the microtensile bond strength (µTBS) testing were performed by a single trained operator. Briefly, the teeth were sectioned parallel to the occlusal surface using a low-speed water-cooled diamond saw (Microremet, Remet, Casalecchio di Reno, Italy) to obtain a 4-mm-thick mid-coronal dentin crown section. The exact dimension was checked with a digital caliper (± 0.01 mm) and the absence of enamel remnants was verified under a stereomicroscope at ×20 magnification. Successively, a standardized smear layer was created on each dentin surface with #240-grit wet silicon-carbide (SiC) paper. The dentin surface was rinsed with water and gently dried with oil-free air flow for 5 s, being careful not to desiccate the specimens.

Dentin blocks were randomly and equally assigned to one of the following groups according to the surface pre-treatment (Riva Star or Riva Star Aqua, SDI) and etching mode (etch-and-rinse, ER, or self-etch, SE) of the universal adhesive (Zipbond Universal, SDI) used for bonding procedures (n = 10):

G1) Zipbond Universal used in the SE mode (control SE group, ZSE);
G2) Riva Star Step 1 (SDF) & Step 2 (KI) applied before ZSE;
G3) Riva Star Aqua Step 1 (AgF) & Step 2 (KI) applied before ZSE;
G4) Zipbond used in the ER mode (control ER group, ZER);
G5) Riva Star Step 1 (SDF) & Step 2 (KI) applied before ZER;
G6) Riva Star Aqua Step 1 (AgF) & Step 2 (KI) applied before ZER.

All the materials were used following the manufacturer’s instructions, as illustrated in Table 1. In experimental groups, the two-step silver-containing solutions were applied to the dentin surface before the application of the universal adhesive and, in the ER mode, after etching (Super Etch, SDI). A 4-mm-thick build-up of composite resin (Luna, A2 shade, SDI) was created on each tooth. A light-emitting diode curing lamp (LED, Elipar DeepCure-L, 3M ESPE, St Paul, MN, USA; irradiance: 1,470 mW/cm^2^; wavelength: 430–480 nm) was used for the polymerization. Finally, the resin–dentin specimens were stored in water at 4°C for 24 h.

**Table 1:** Names, compositions, and application mode of the materials used in the study (information supplied by the manufacturer)

Material	Composition	Mode of use
Riva Star	Step 1: silver, fluoride, ammonia Step 2: potassium iodide (KI)	Step 1: Dispense one drop of silver-containing solution on a dappen dish. Carefully apply the solution for 10 s to treatment site only using a medium sized microbrush. Step 2: Immediately after, dispense two drops of KI solution onto fresh dappen dish. Apply a generous amount of the solution to the treatment site using a medium sized microbrush until the creamy white precipitate turns clear. Wash thoroughly with water for at least 10 s and air-dry for 10 s.
Riva Star Aqua	Step 1: silver, fluoride, water Step 2: potassium iodide (KI)	Follow instructions as for Riva Star.
Zipbond Universal	Adhesive monomers including 10-methacryloyloxydecyl dihydrogen phosphate (MDP), ethanol, water, fluoride	Dispense 1-2 drops into a mixing well. Scrub onto tooth tissues with a brush for 10 s. Wait 5 s. Blow with oil-free air until no movement of the adhesive (no less than 5 s). Light-cure for 10 s (460–480 nm wavelength) with LED curing light.
Super etch syringe	37% phosphoric acid	Dry the surface to be etched. Etch for 10 s, then rinse with water for 10 s and air-dry for 10 s.
Luna (A2 shade)	22.5% wt (39% vol.) multifunctional methacrylic ester, 77.5% wt (61% vol.) inorganic filler (40 nm – 1.5 μm)	Place composite in increments of 2 mm or less. Light-cure each increment for 20 s for light shade with a LED curing device.
All the materials used in this study were produced by the same manufacturer (SDI, Bayswater VIC, Australia)

After storage, the specimens were cut perpendicularly to the adhesive interface into resin–dentin sticks, with a cross-sectional surface area of ~0.9 mm^2^, using a low-speed water-cooled diamond saw (Microremet, Remet), following the non-trimming technique of the µTBS test.^43^ The sticks of each group were randomly and equally divided and immersed in artificial saliva at 37°C for either 24 h (T0) or 2 years (T2). During the 2-year storage period, the artificial saliva was renewed every 2 weeks.^44^

Prior to testing, each stick was measured using a digital caliper (±0.01 mm). The ends of the sticks were fixed to the test block of a µTBS testing machine (Bisco, Schaumburg, IL, USA) using cyanoacrylate glue. Each stick was stressed to failure at a crosshead speed of 1 mm/min. The force required to fracture the specimens was recorded in Newton (N) and converted to stress (MPa) using the equation: bond strength (MPa) = force (N)/bonded surface area (mm^2^).

After testing, the fractured sticks were examined by a single blind trained observer under a stereomicroscope at 50× magnification to determine the type of failure. Failures were classified as follows: adhesive (A, along the dentin/adhesive interface), cohesive in dentin (CD, within the dentin surface), cohesive in composite (CC, within the composite resin) or mixed (M, adhesive and cohesive failures occurred simultaneously).

### Scanning Electron Microscope (SEM) Evaluation and Energy Dispersive Spectroscopy Analysis (EDS)

Two fractured resin–dentin sticks per group, each with bond strength values close to the group mean, were further examined using a scanning electron microscope (SEM, Mira3, Tescan, Brno, Czech Republic). The sticks were first immersed in a 2.5% glutaraldehyde in 0.1% cacodylate buffer and then dehydrated through ascending ethanol solutions (50%, 70%, 80%, 90%, 95%, 100%) and HMDS. The dentin side of the fractured sticks was mounted on metal stubs and gold-palladium sputter-coated before evaluation at different magnifications (200× and 2.000×, accelerating voltage of 10.00 kV).

Additionally, EDS was performed on the entire dentin portion of the fractured specimens at T0 and T2 to study the chemical profile.

### In-situ Zymography Analysis

To assess the dentin MMPs’ activity, additional freshly extracted sound human molars (n = 3) were collected for *in-situ* zymography, conducted by the same aforementioned calibrated operator following a previously validated protocol.^33^ Adhesive procedures and aging were performed as previously described for the µTBS test.

At T0 and T2, 2 resin–dentin specimens from each tooth per group were affixed to a microscope glass slide with cyanoacrylate glue, ground down, and progressively polished with wet SiC papers (#600-, #1,200-, #4,000-grit) to obtain ~50 µm-thick sections.

A 50-µL quantity of self-quenched fluorescein-conjugated gelatin (E-12055; Molecular Probes, Eugene, OR, USA) was placed on top of each polished specimen, covered with a coverslip, and incubated in a dark, humidified chamber at 37°C overnight.

After incubation, the specimens were observed under a multi-photon confocal laser scanning microscope (Leica SP8, Leica Microsystems, Wetzlar, Germany; excitation/emission wavelength: 488/530 nm). Several z-stack images (~15 μm-thick, one image for each 1 μm into the depth of the sample) of the HL were made per specimen. The hydrolysis of the gelatin substrate, indicative of endogenous gelatinolytic enzyme activity, was quantified as the integrated density of the fluorescence signals for each image using ImageJ software (National Institutes of Health, Bethesda, MD, USA). Differences in fluorescence intensity between the tested groups were used as a relative measurement of the differences in enzymatic activity within the HL.

### Statistical Analysis

The data retrieved from the µTBS test and the *in-situ* zymography analysis were tested for normal distribution and homoscedasticity (Shapiro–Wilk and Brown–Forsythe test; P >0.05). Accordingly, the three-way analysis of variance (three-way ANOVA) and all pairwise multiple comparison procedures (Holm–Sidak method) were conducted to investigate the effect of three variables – “pre-treatment” (Control, Riva Star or Riva Star Aqua); “etching mode” (ER and SE); and “aging” (T0 and T2) – and their interaction on bond strength and endogenous enzymatic activity. Additionally, one-way ANOVA was performed to examine the differences between the different groups. The statistician who performed the analysis was unaware of the group’s belonging.^14^ For all tests, the level of statistical significance was set at P <0.05 (SigmaPlot 15.0, Systat Software, San Jose, CA, USA).

## RESULTS

### Microtensile Bond Strength (µTBS) Test

The mean and standard error of µTBS values (expressed as MPa), before and after aging in artificial saliva, along with the statistical differences/similarities between the groups retrieved from the one-way ANOVA test, are presented in Table 2. Three-factor ANOVA revealed that the variables “pre-treatment,” “etching mode,” and “aging” significantly influenced bond strength values (P <0.05), while no statistically significant interactions between the variables were detected (P >0.05).

**Table 2:** Means ± standard errors of µTBS values (expressed as MPa)

	T0	T_2_
SE	ER	SE	ER
Control	33.7±1.6 ^A,b^ [11CC, 68M, 21A]	42.1±1.9 ^A,a^ [17CC, 40M, 38A, 5CD]	15.0±2.4 ^A,c^ [57M, 43A]	21.1±2.4 ^A,c^ [12CC, 70M, 18A]
Riva Star	24.8±2.5 ^AB,b^ [8CC, 40M, 40A, 12CD]	35.6±1.4 ^AB,a^ [8CC, 48M, 37A, 7CD]	12.9±1.4 ^A,c^ [67M, 33A]	16.9±1.4 ^A,bc^ [4CC, 52M, 44A]
Riva Star Aqua	24.2±2.3 ^B,b^ [59M, 41A]	33.0±2.2 ^B,a^ [15CC, 65M, 18A, 2CD]	11.8±1.5 ^A,c^ [88M, 12A]	14.1±1.7 ^A,bc^ [84M, 16A]
T0 and T2 indicate specimens that were tested after storage in artificial saliva at 37 °C for 24 h or 2 years, respectively. Different superscript uppercase letters indicate statistically significant differences (P <0.05) within the columns. Different superscript lowercase letters indicate statistically significant differences (P <0.05) within the rows. Distribution of failure modes among tested groups is also reported in percentages in square brackets and classified as follows: CC: cohesive in composite resin; M: mixed; A: adhesive; CD: cohesive in dentin

Experimental groups exhibited a significantly lower bond strength compared to control groups (P <0.05), while no significant difference was reported between Riva Star and Riva Star Aqua groups (Control > Riva Star = Riva Star Aqua; P >0.05). ER groups demonstrated significantly higher bonding values compared to SE groups (TE>SE; P <0.05). Two-year aging in artificial saliva negatively influenced bond strength among all groups (T0 > T2; P <0.05).

The failure mode analysis of the tested specimens revealed a predominance of mixed fractures across all groups, irrespective of pre-treatment, etching, or aging (Table 2).

### Scanning Electron Microscope (SEM) Evaluation and Energy Dispersive Spectroscopy (EDS) Analysis

**Fig 1:**
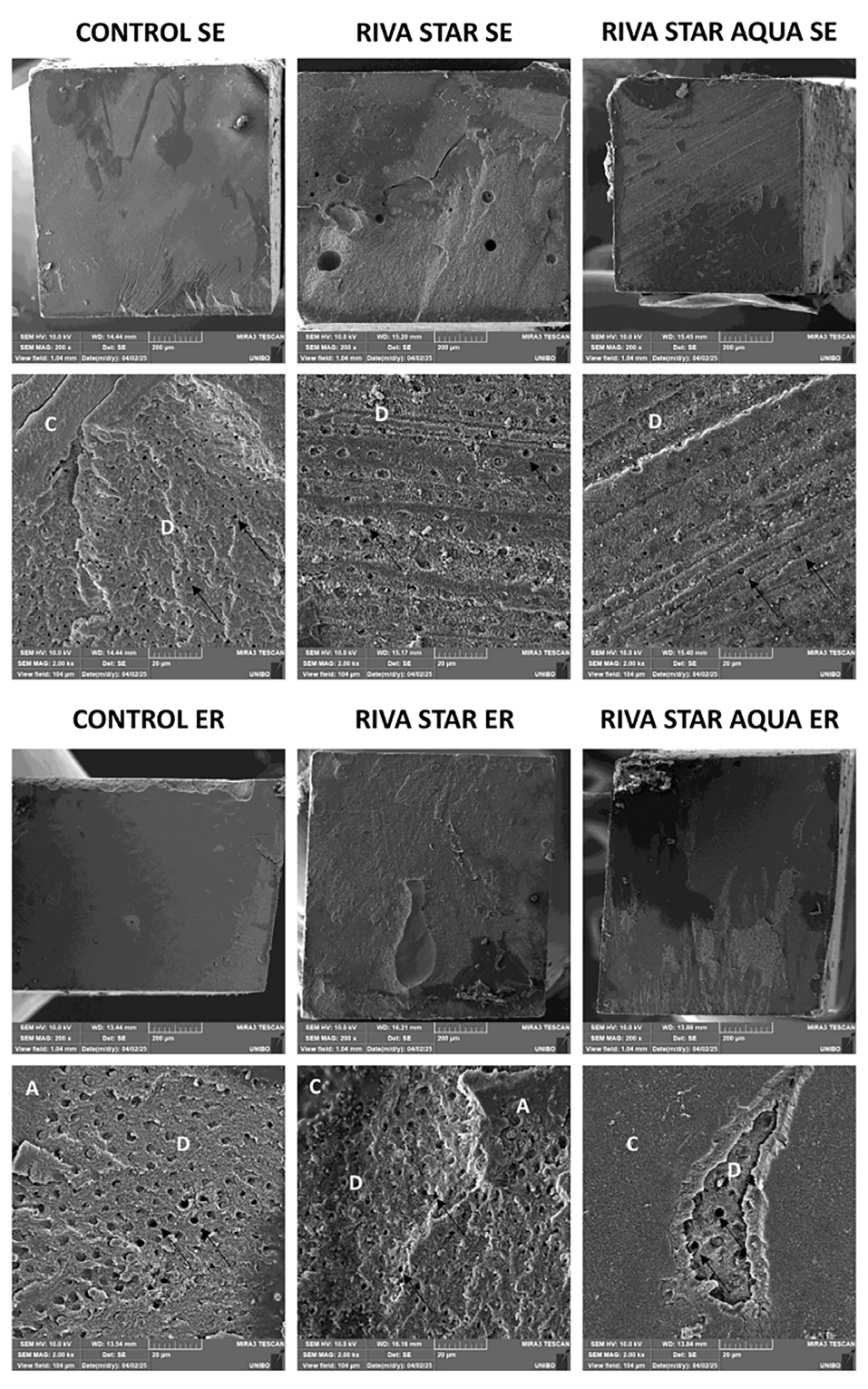
SEM images of fractured microtensile sticks at T0 (200× magnification on the upper image and 2,000× on the lower image for each group). D: dentin; A: adhesive; C: composite; black arrows: dentinal tubules. Observations were conducted on representative fractured specimens registering bonding values close to the mean of each group, as follows indicated (values expressed in MPa): control SE 32.3; Riva Star SE 25.1; Riva Star Aqua SE 25.4; control ER 41.3; Riva Star ER 34.5; Riva Star Aqua ER 31.7.

**Fig 2:**
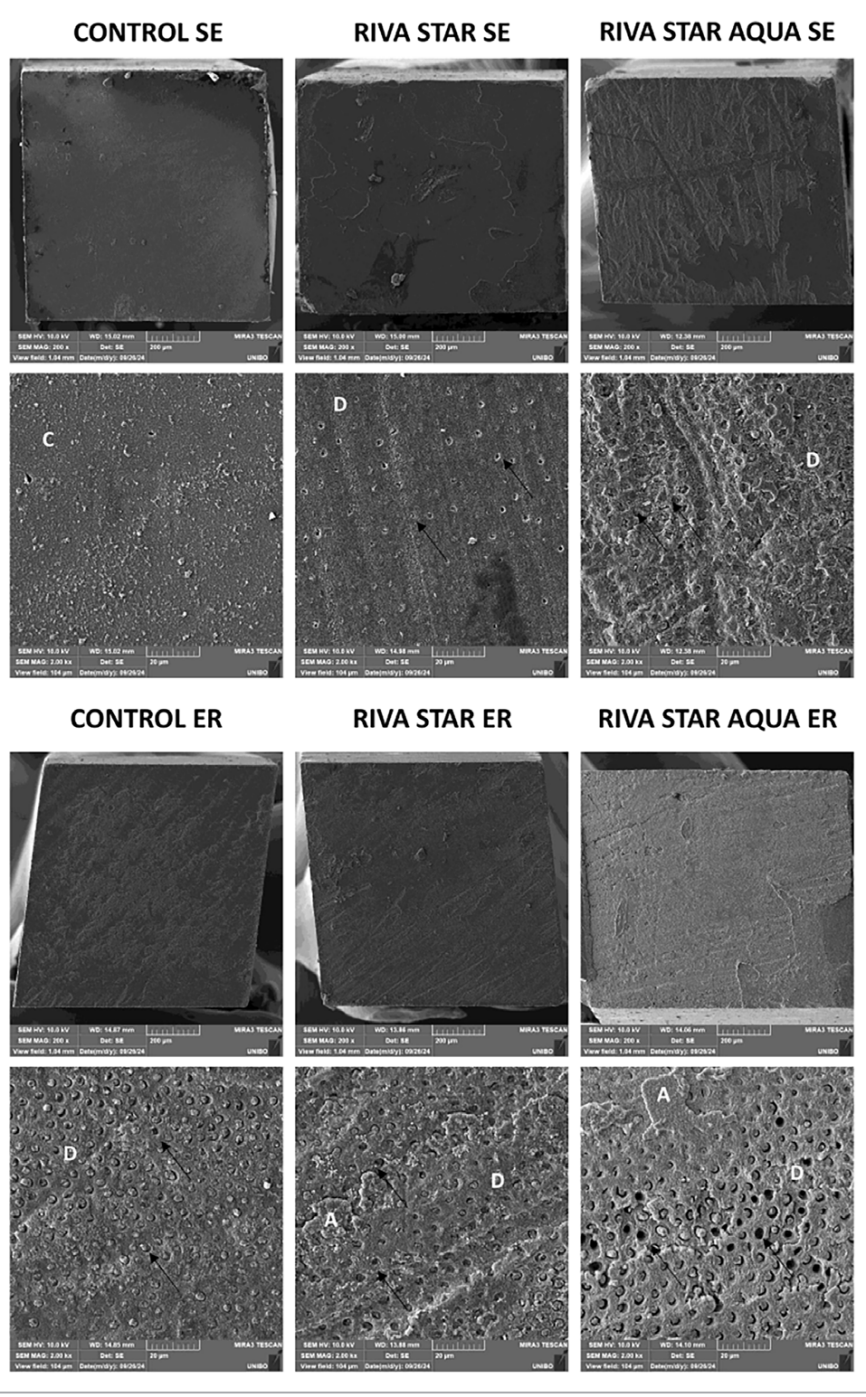
SEM images of fractured microtensile sticks at T2 (200× magnification on the upper image and 2,000× on the lower image for each group). D: dentin; A: adhesive; C: composite; black arrows: dentinal tubules. Observations were conducted on representative fractured specimens registering bonding values close to the mean of each group, as follows indicated (values expressed in MPa): control SE 15.4; Riva Star SE 14.1; Riva Star Aqua SE 12.6; control ER 21.3; Riva Star ER 15.1; Riva Star Aqua ER 13.6.

Representative SEM fractographic images illustrating the failure patterns are displayed in Figures 1 and 2. In SE groups, the collagen fibers and dentin tubules were mostly covered by the composite resin and the smear layer, at both T0 and T2. Conversely, in ER groups, increased smear layer removal was observed at both T0 and T2, with exposed intratubular collagen fibers and dentinal tubules visibly open or partially infiltrated by the resin.

**Fig 3:**
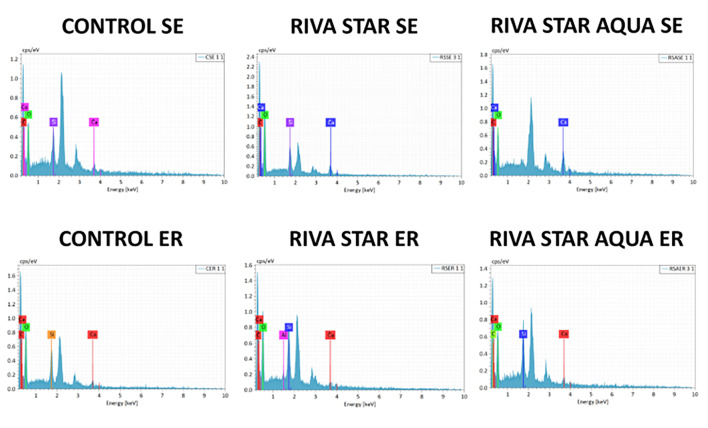
EDS analysis of fractured microtensile sticks at T0 (magnification 200×).

**Fig 4:**
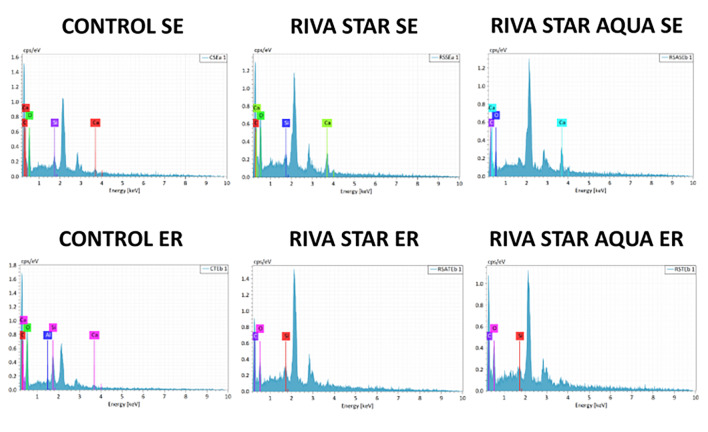
EDS analysis of fractured microtensile sticks at T2 (magnification 200×).

The EDS elemental analyses for chemical characterization of particles on the fractured adhesive interface at T_0_ and T_2_ are presented in Figure 3 and Figure 4, respectively. Regardless of etching and aging, the most prevalent chemical elements detected in control groups were carbon, calcium, oxygen, and silicon. In experimental groups, no silver was detected on the bonding interfaces of specimens treated with Riva Star and Riva Star Aqua at T_0_ and T_2_.

### In-situ Zymography Analysis

**Fig 6a and b:**
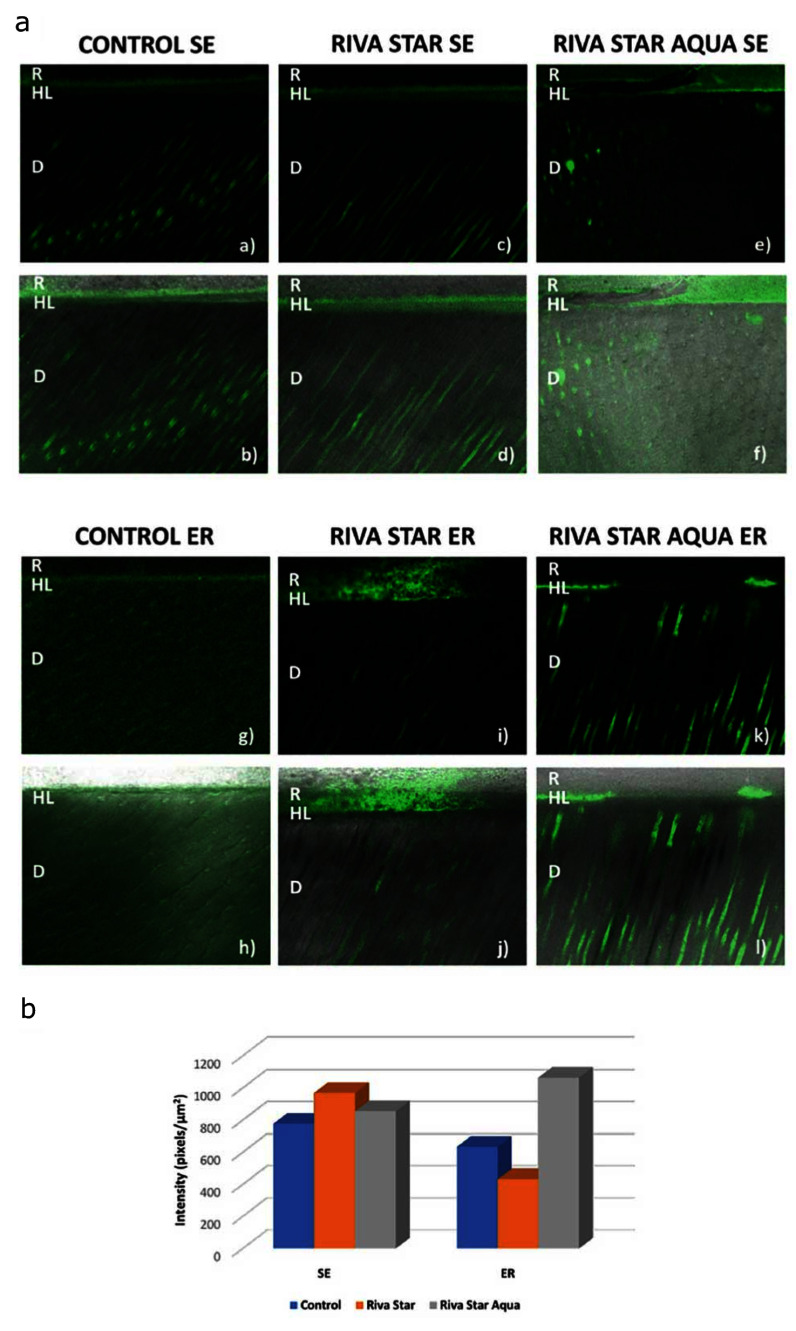

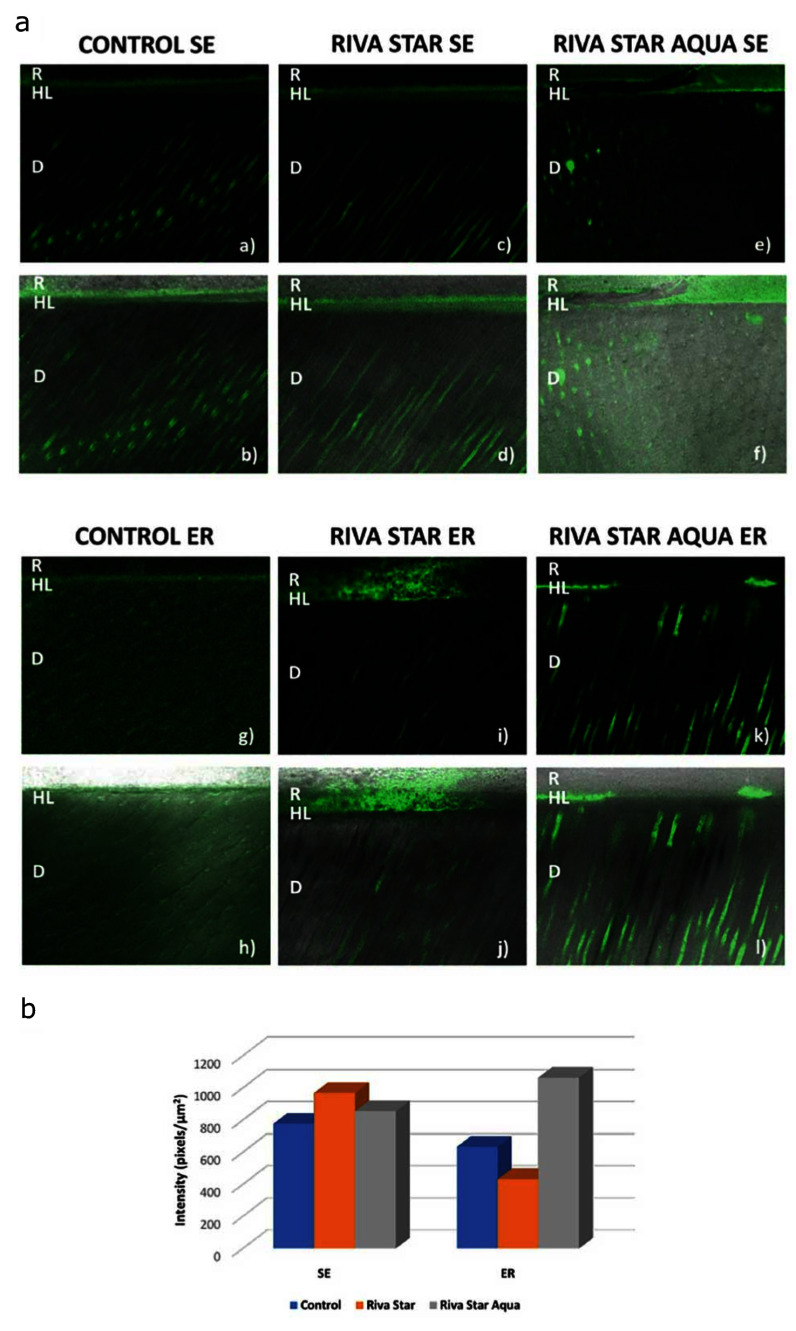
*In-situ* zymography results obtained after 1 year of aging in artificial saliva at 37°C (T2). (a) Representative examples of the resin-dentin interfaces incubated with fluorescent gelatin. Images acquired in the green channel (a, c, e, g, i, k). Composite images obtained by merging the differential interference contrast image and those obtained in the green channel (b, d, f, h, j, l). D: dentin; HL: hybrid layer; R: composite resin. (b) Bar graph representing gelatinolytic activity, expressed as the intensity of green fluorescence (pixels/µm^2^) within the HL.

Representative confocal laser scanning microscopy images showing the level of endogenous enzymatic activity as the density of the green fluorescence signal are displayed in Figure 5a and Figure 6a. Confocal laser scanning microscopic examination of *in-situ* zymography specimens in all groups detected green fluorescence within the HL, as well as underlying dentinal tubules.

Three-factor ANOVA revealed that the variable “pre-treatment” significantly influenced endogenous enzymatic activity (P <0.05), while no significant difference was reported by the variables “etching mode” and “aging” (P >0.05). Furthermore, all the interactions between the three investigated variables were statistically significant (P <0.05).

The mean results of endogenous gelatinolytic activity, expressed as the integrated density of the green fluorescence signal within the HL, are summarized in Figure 5b and Figure 6b. Riva Star Aqua groups exhibited a significantly higher dentin gelatinolytic activity within the HL compared to control and Riva Star groups (P <0.05). Notably, fluorescence density level was more expressed in control groups compared to Riva Star groups, irrespective of the etching mode and aging period (P <0.05).

## DISCUSSION

The present study aimed to investigate the influence of two-step silver-containing solutions, differing in solvent composition, on bonding performance and endogenous enzymatic activity of a universal adhesive to sound dentin after 2 years of artificial aging. Our results demonstrated that Riva Star and Riva Star Aqua, applied as sound dentin pre-treatments, significantly reduced µTBS values and increased (Riva Star Aqua) or reduced (Riva Star) dentin gelatinolytic activity within the HL compared to controls. Therefore, the first and second working hypotheses of the study were accepted. Additionally, artificial aging affected bond strength, but not MMPs activity; thus, the third working hypothesis was only partially accepted.

Based on our findings, the investigated two-step silver-containing solutions decreased bond strength compared to the control, even if a rinsing step was performed before bonding procedures. These results are in accordance with other studies where 38% silver-fluoride solutions (such as Step 1 of the materials investigated in the article) alone^32,30,34,28,24,46,49^ or followed by KI application^1,12,16,29,50^ negatively influenced bonding performance. Such an effect could be due to the chemical interaction of silver-containing solutions with dentin. Namely, these products react with hydroxyapatite to produce calcium fluoride (CaF2) and silver phosphate (Ag3PO4). These precipitates on the surface and within dentin tubules^53^ may potentially influence the interaction of dentin with the adhesive resin on several levels. Mechanically, the precipitates might hinder adhesive infiltration and penetration, impairing the quality of the interface and reducing bonding performance.^10,21,24,29,32^ Furthermore, universal adhesives enhance hydrolysis resistance and bond durability through functional monomers, such as 10-methacryloyloxydecyl dihydrogen phosphate (10-MDP),^26^ which chemically interact with hydroxyapatite to form Ca-10-MDP salts in a process known as nanolayering.^8,11^ In the present study, a universal adhesive containing 10-MDP was used following the application of Riva Star and Riva Star Aqua in both SE and ER modes. Our findings suggest that the silver-containing solutions might compete with the 10-MDP for calcium sites, hindering the chemical interaction of the functional monomer with the substrate and the subsequent nanolayering formation within the HL,^29^ resulting in the reduced bond strength values observed.

The existing evidence related to silver-containing products is fairly discordant and some authors have observed no negative effect on the bond strength of composite resin restorations to sound dentin.^2,13,18,45,47^ Given that all the aforementioned studies cited relate to products with the same concentration of active ingredient (38% silver fluoride) applied to sound dentin before composite restoration, the inconsistency among the results might be attributed to other factors. The latter includes the extreme variability of the application protocol,^54^ the presence of different excipients among the products of different manufacturers,^10^ and the type and application modes of adhesive systems.^5,6^ For instance, recent literature has emphasized the critical issue of product application due to its high sensitivity, proposing a thorough rinsing step after surface pre-treatment to preserve bond strength.^20^

Regarding the application mode, ER groups demonstrated significantly higher bonding values compared to SE groups. These findings align with other studies on silver-containing solutions, where the ER approach exhibited higher bond strength of composite restorations compared to the SE approach.^24,45^ It may be hypothesized that SE mode may be negatively affected by the alkaline nature of silver-containing solutions (particularly high in Riva Star), which may interfere with the function of acidic monomers usually present in universal adhesives, thereby hampering the formation of the HL.^24^ Moreover, the persistence of silver-phosphate deposits even after the rinsing step may further jeopardize the bonding process.^34^ Despite the differences µTBS results between ER and SE groups, a predominance of mixed failures in the resin–dentin interface was recorded regardless of etching mode.

The SEM/EDS analysis demonstrated that, regardless of etching and aging, control groups exhibited the expected presence of carbon, calcium, oxygen, and silicon, consistent with previous research.^12^ Notably, no silver was detected either in Riva Star or Riva Star Aqua groups at baseline and after 2 years of artificial aging, regardless of whether the fractured interface exposed dentinal tubules or was predominantly covered by composite resin. In contrast, previous research reported the presence of silver on the adhesive surface^12^ or inside the dentinal tubules,^22,24,27,4^6 even when there were seemingly no precipitates on the surface. Thus, it may not be excluded that silver traces were present deep inside the tubules, not detected by EDS surface analysis. Future studies are warranted to further investigate the penetration depth of the tested solutions.

In terms of MMPs’ activity, the present study found that the water-based Riva Star Aqua exhibited the highest fluorescence density level within the HL, while the ammonia-based Riva Star demonstrated a superior inhibitory effect on MMPs compared to controls. Several studies have corroborated the application of 38% silver fluoride as an inhibitor of dentin collagen degradation.^15,37–39,56^ It is well established that both etching and adhesive application are responsible for the activation of bacterial collagenases^35^; thus, it can be hypothesized that the strong alkalinity of the ammonia-based solution may limit the proteolytic activities of collagenases.^4^ In contrast, the water-based Riva Star Aqua, with its near-neutral pH and higher water content, may have induced MMP-mediated degradation.^12^ Regarding the inconsistency between µTBS test and in-situ zymography findings, it is important to highlight that the durability of the resin-dentin interface is influenced not only by endogenous enzymatic activity but also by resin hydrolysis and the aforementioned impairment of hybridization of dentin with the adhesive resin.^31^ Thus, as suggested in a previous article,^12^ the application of two-step silver-containing solutions and/or storage in artificial saliva may have detrimental effects on HL.

Despite the potential benefits of two-step silver-containing solutions as therapeutic agents, some aspects remain to be considered. The results of the current study indicate that Riva Star inhibits MMPs. However, given that complete coverage of the bonding surface with two-step silver-containing solutions significantly impairs adhesion to sound dentin, it remains to be confirmed whether a selective application limited to the cavity floor may represent a more favorable strategy, potentially reducing the risk of premature failure of composite restorations.^29,34^ Moreover, further in-vitro and in-vivo studies are warranted to standardize the application protocol, investigate the mechanisms of resin penetration when silver-containing solutions are used, and comprehensively evaluate the effects of the latest introduced product, Riva Star Aqua.

## CONCLUSION

Dentin pretreatments with the two-step silver-containing solutions, Riva Star and Riva Star Aqua, demonstrated an adverse effect on the dentin bond strength of composite resin restorations placed with a universal adhesive. The ammonia-based product Riva Star exhibited a greater inhibitory effect on MMPs.

### Clinical Relevance

The use of two-step silver-containing solutions as dentin pre-treatment may affect the longevity of bonded composite resin restorations.

### Acknowledgments

The Confocal Laser Scanning microscope is available at the imaging facility Centro Interdipartimentale Grandi Strumenti (CIGS) of the University of Modena and Reggio Emilia. We are grateful to SDI for partially providing the products used in this study.

This study was supported by PNRR - M4C2 – Investimento 1.1 “Progetti di Ricerca di Rilevante Interesse Nazionale (PRIN)” as part of the project: M.E.T.A.D.E.N.T.I – Mih-affected teeth: crossTalk between genetic background and environmental influences – funded by the European Union – Next Generation EU. Project number MUR: 20222YTC5A – CUP: D53D23007740006

**Fig 5a and b:** *In-situ* zymography results obtained after 24 h of aging in artificial saliva at 37°C (T0). (a) Representative examples of the resin-dentin interfaces incubated with fluorescent gelatin. Images acquired in the green channel (a, c, e, g, i, k). Composite images obtained by merging the differential interference contrast image and those obtained in the green channel (b, d, f, h, j, l). D: dentin; HL: hybrid layer; R: composite resin. (b) Bar graph representing gelatinolytic activity, expressed as the intensity of green fluorescence (pixels/µm^2^) within the HL.
